# A Refined, Controlled 16S rRNA Gene Sequencing Approach Reveals Limited Detection of Cerebrospinal Fluid Microbiota in Children with Bacterial Meningitis

**DOI:** 10.1128/spectrum.00361-23

**Published:** 2023-05-04

**Authors:** Christopher E. Pope, Kathryn B. Whitlock, Paul Hodor, David D. Limbrick, Patrick J. McDonald, Jason Hauptman, Lucas R. Hoffman, Tamara D. Simon

**Affiliations:** a Department of Pediatrics, University of Washington, Seattle, Washington, USA; b New Harmony Statistical Consulting, Clinton, Washington, USA; c Seattle Children's Research Institute, Seattle, Washington, USA; d Department of Neurosurgery, Washington University in St. Louis, St. Louis, Missouri, USA; e St. Louis Children’s Hospital, St. Louis, Missouri, USA; f Division of Neurosurgery, University of British Columbia, Vancouver, British Columbia, Canada; g British Columbia Children’s Hospital, Vancouver, British Columbia, Canada; h Department of Neurosurgery, University of Washington, Seattle, Washington, USA; i Seattle Children's Hospital, Seattle, Washington, USA; j Department of Pediatrics, University of Southern California, Los Angeles, California, USA; k The Saban Research Institute, Los Angeles, California, USA; l Children’s Hospital Los Angeles, Los Angeles, California, USA; University of California, San Diego

**Keywords:** bacterial meningitis, cerebrospinal fluid, high-throughput sequencing

## Abstract

Advances in both laboratory and computational components of high-throughput 16S amplicon sequencing (16S HTS) have markedly increased its sensitivity and specificity. Additionally, these refinements have better delineated the limits of sensitivity, and contributions of contamination to these limits, for 16S HTS that are particularly relevant for samples with low bacterial loads, such as human cerebrospinal fluid (CSF). The objectives of this work were to (i) optimize the performance of 16S HTS in CSF samples with low bacterial loads by defining and addressing potential sources of error, and (ii) perform refined 16S HTS on CSF samples from children diagnosed with bacterial meningitis and compare results with those from microbiological cultures. Several bench and computational approaches were taken to address potential sources of error for low bacterial load samples. We compared DNA yields and sequencing results after applying three different DNA extraction approaches to an artificially constructed mock-bacterial community. We also compared two postsequencing computational contaminant removal strategies, decontam R and full contaminant sequence removal. All three extraction techniques followed by decontam R yielded similar results for the mock community. We then applied these methods to 22 CSF samples from children diagnosed with meningitis, which has low bacterial loads relative to other clinical infection samples. The refined 16S HTS pipelines identified the cultured bacterial genus as the dominant organism for only 3 of these samples. We found that all three DNA extraction techniques followed by decontam R generated similar DNA yields for mock communities at the low bacterial loads representative of CSF samples. However, the limits of detection imposed by reagent contaminants and methodologic bias precluded the accurate detection of bacteria in CSF from children with culture-confirmed meningitis using these approaches, despite rigorous controls and sophisticated computational approaches. Although we did not find current DNA-based diagnostics to be useful for pediatric meningitis samples, the utility of these methods for CSF shunt infection remains undefined. Future advances in sample processing methods to minimize or eliminate contamination will be required to improve the sensitivity and specificity of these methods for pediatric meningitis.

**IMPORTANCE** Advances in both laboratory and computational components of high-throughput 16S amplicon sequencing (16S HTS) have markedly increased its sensitivity and specificity. These refinements have better delineated the limits of sensitivity, and contributions of contamination to these limits, for 16S HTS that are particularly relevant for samples with low bacterial loads such as human cerebrospinal fluid (CSF). The objectives of this work were to (i) optimize the performance of 16S HTS in CSF samples by defining and addressing potential sources of error, and (ii) perform refined 16S HTS on CSF samples from children diagnosed with bacterial meningitis and compare results with those from microbiological cultures. We found that the limits of detection imposed by reagent contaminants and methodologic bias precluded the accurate detection of bacteria in CSF from children with culture-confirmed meningitis using these approaches, despite rigorous controls and sophisticated computational approaches.

## INTRODUCTION

Cerebrospinal fluid (CSF) is generally considered to be sterile. When infection is clinically suspected, the identification of microorganisms in CSF has traditionally relied upon conventional bacterial cultures, which are tailored to identify specific human pathogens. Our earlier work focused on children with hydrocephalus requiring CSF shunts, in whom CSF infection is generally thought to involve specific microbes adherent to shunt hardware and that are, due to their physiological states, variably detectable by culture. Indirect evidence suggests that CSF shunt infections commonly involve microbes that are not detected by conventional bacterial cultures but that can be identified by molecular microbiological tools.(1) We previously used two molecular microbiological tools—high-throughput 16S amplicon sequencing (16S HTS) and quantitative PCR (qPCR)—to characterize the DNA of culturable and nonculturable microbes to detect (2) and quantify total CSF bacterial loads (3) in CSF from children with clinically diagnosed shunt infections. These earlier studies detected low CSF levels of DNA from diverse bacteria and fungi that were not detected by conventional bacterial culture.

Since then, advances in both the laboratory and computational components of these molecular microbiological approaches have markedly increased both sensitivity and specificity of both these approaches, especially 16S HTS. These refinements have also better delineated the limits of sensitivity, and contributions of contamination to these limits, for 16S HTS that are particularly relevant for samples with low bacterial loads, such as human CSF. For example, reagents used for 16S HTS are now known to commonly introduce contaminating bacterial DNA, as well as bias for or against specific microbial taxa, which can either obscure the contribution of truly infecting microbes or erroneously identify contaminating bacteria in human infection samples.(4) These experiences have identified specific methodologic refinements, including laboratory and computational controls and systematic bench techniques, that are now in broad use to distinguish true sample microbiota from experimental error, to rigorously exclude contaminants, to delineate and improve the limits of detection, and to define clinical utility.

For sample types with high bacterial loads, such as fecal samples, the contributions of the confounders described above are comparably minor. However, in the context of low bacterial load samples, these details can significantly impact accuracy (5). There are three known main sources of error in microbiota analysis of low-load samples, each of which requires the inclusion of specific controls to address them: (i) contamination of DNA extraction kits and PCR reagents with bacteria and/or free DNA (reagent contamination) (6); (ii) differences in DNA extraction efficiency for different bacterial species (extraction bias) (7); and (iii) variation of DNA extraction results, especially from samples with low bacterial load, according to batch of reagents used and from day to day (batch effect) (6, 8, 9).

The objectives of this work were to (i) optimize the performance of 16S HTS in CSF samples with low bacterial loads by defining and addressing potential sources of error (including the contributions of reagent contamination, variable DNA extraction efficiency and bias, and batch effect), and (ii) perform refined 16S HTS on CSF samples obtained from children diagnosed with bacterial meningitis and then compare the results with those from microbiological cultures. We hypothesized that, unlike with CSF shunt infection, 16S HTS controlling for contaminants and bias would identify relatively higher abundances of bacteria in the CSF from children with bacterial meningitis. We also hypothesized that the relative abundances of known pathogenic organisms identified by 16S HTS would be higher than those of organisms not known to be CNS pathogens in meningitis samples.

## RESULTS

### A bench and computational pipeline to define and address contributions of contaminants and bias to 16S HTS for pediatric CSF samples.

**FIG 1:**
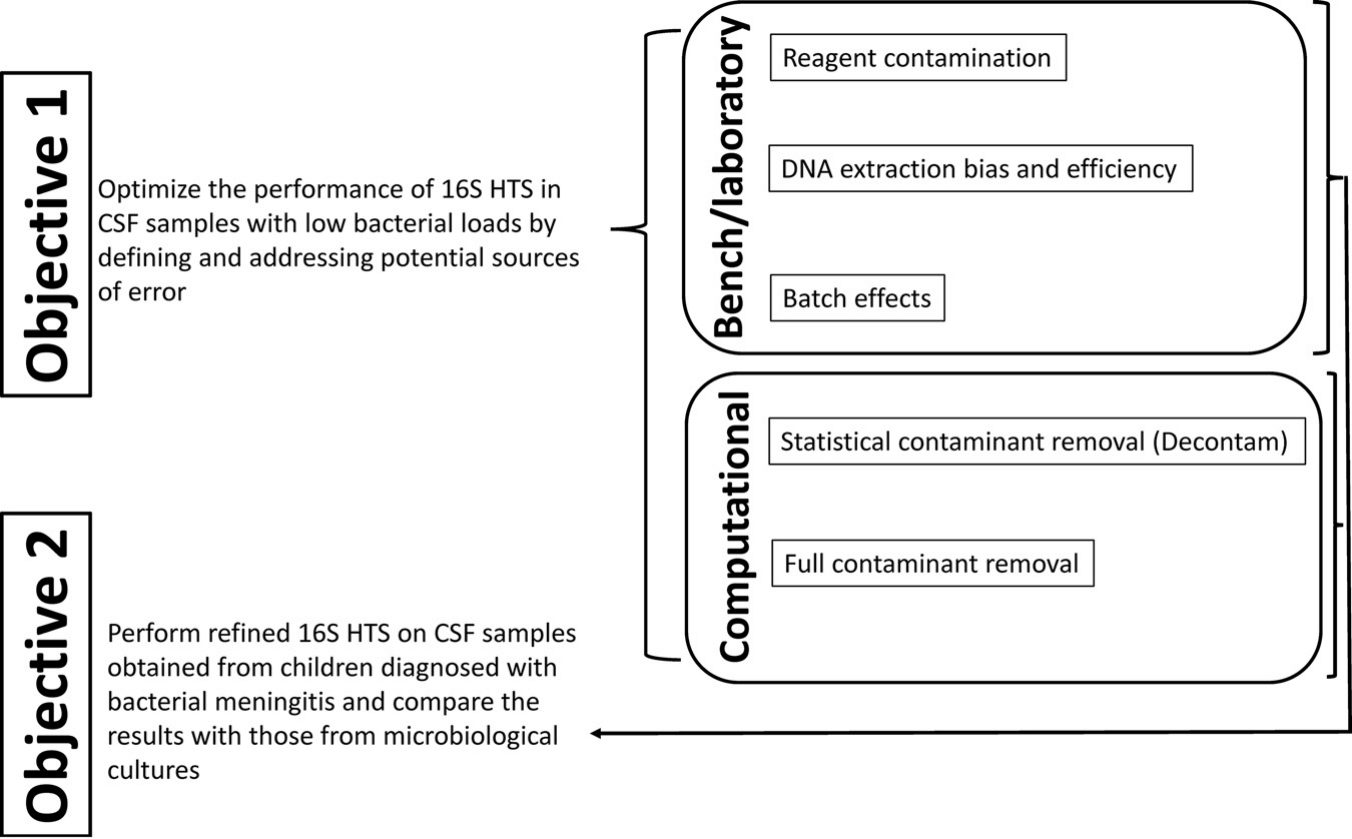
Schematic of study objectives. Bench and computational approaches were refined using mock communities and robust negative controls to define and address contributions of contaminants and bias to 16S HTS in objective 1. These bench and computational pipelines were applied to CSF samples from children with meningitis in objective 2.

As described above and shown in Fig. 1, we used several laboratory methodological techniques to address and control for potential sources of systematic error in defining the microbiota of samples with low bacterial load, specifically due to reagent contamination and DNA extraction efficiency and bias, and batch effects.

**FIG 2:**
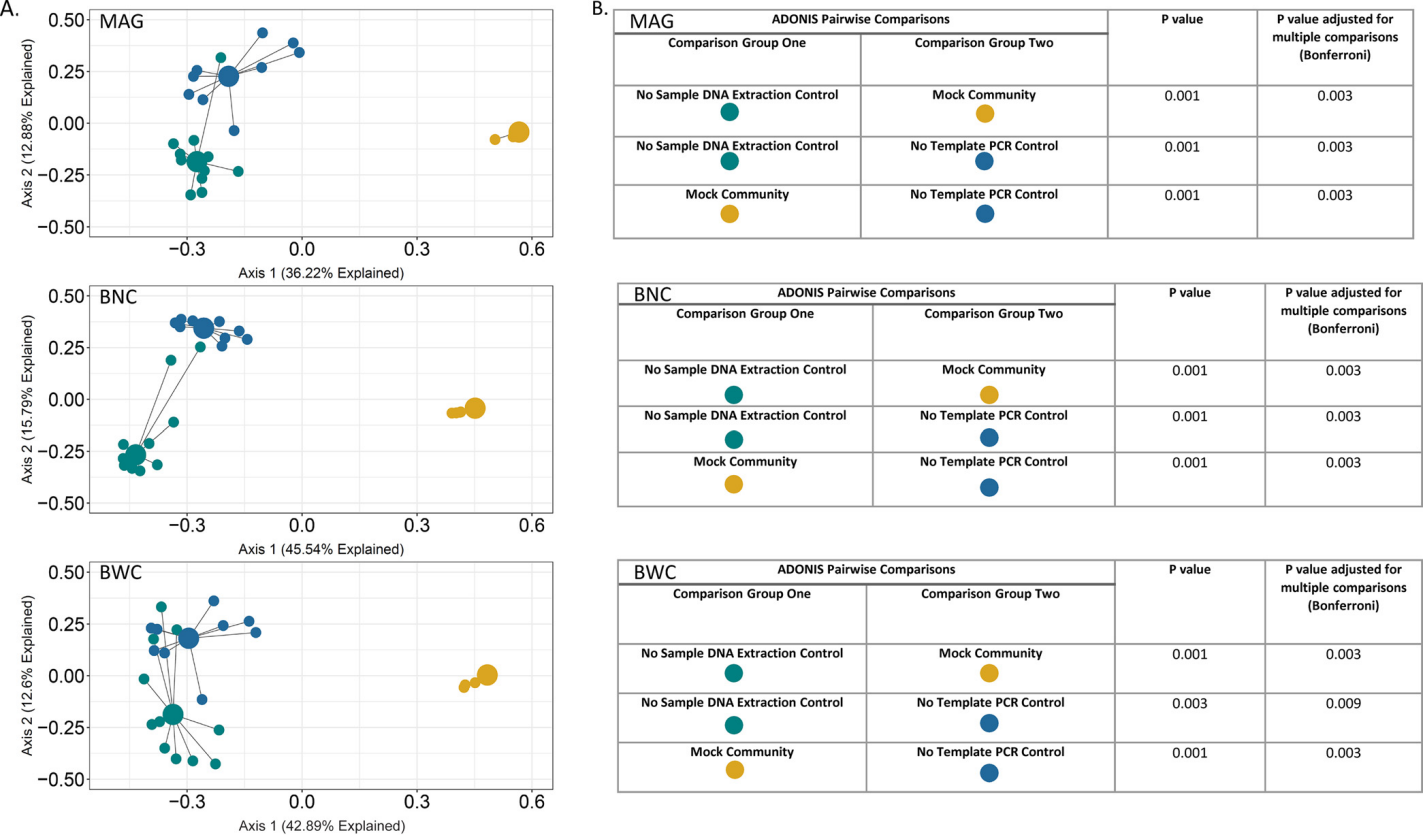
PCoA of mock community and negative-control samples using 3 different DNA extraction methods. (A) PCoA plot of samples using Bray–Curtis distances based on sequence read composition. The PCoA describes relationships between taxonomic composition patterns of the individual samples and sample type groupings. Individual samples are represented by the small dots, colored by sample type. Large dots represent the centroid of each group, with lines connecting individual sample dots to their respective centroids. Centroids represent the averages of the taxonomic composition of each sample type. (B) Data tables of ADONIS pairwise comparisons within DNA extraction method groupings. Comparisons were between mock communities and their no-sample extraction controls and no-template library PCR controls.

To quantify bias introduced by reagent contamination as well as extraction techniques, we compared the final microbiota profiles yielded by three different sample DNA extraction methods applied to an artificially constructed mixture of cells of bacterial taxa frequently isolated from CSF shunt infections (Table 1). This mock community then served as a useful control not only for comparing the results of different DNA extraction methods, for but other potential sources of error and bias as noted below. To characterize the contribution of reagent contamination to the HTS-based microbiota profiles of a defined mock community, we generated a principal coordinates analysis (PCoA) plot (Fig. 2) displaying differences in overall sequencing-defined microbiota profiles for the mock community yielded by the three extraction methods, and for the two controls. Mock community profiles were qualitatively similar regardless of extraction technique, but different from those of controls, which reflected reagent contaminants during DNA extraction—“no sample extraction control”—and PCR—“no-template library PCR control”—respectively. The taxonomic constituencies of controls and mock communities differed, and DNA extraction and library construction were performed physically and temporally separate for mock communities and other controls, indicating that taxa identified in controls were not due to cross-contamination from the mock communities. In addition, the profiles of the two control types differed significantly from each other, as expected given these control types represent distinct sources of potential contamination. Therefore, extraction method was not a significant source of bias for this analysis, and the contribution of reagent contaminants to the 16S HTS-based microbiota profiles of a defined mock community was minimal.

**TABLE 1:** Bacterial species and genera present in the CSF mock community

Bacterial species and genera	CFU/μL
Acinetobacter baumannii	5.00E + 06
*Corynebacterium* sp.	5.00E + 06
Cutibacterium acnes	5.00E + 06
Enterococcus faecalis	5.00E + 06
Escherichia coli	5.00E + 06
Klebsiella pneumoniae	5.00E + 06
Proteus mirabilis	5.00E + 06
Pseudomonas aeruginosa	5.00E + 06
Staphylococcus aureus	5.00E + 06
Staphylococcus epidermidis	5.00E + 06
Streptococcus pyogenes	5.00E + 06
Streptococcus salivarius	5.00E + 06
Total CFU equivalents/μL	6.00E + 07

**FIG 3:**
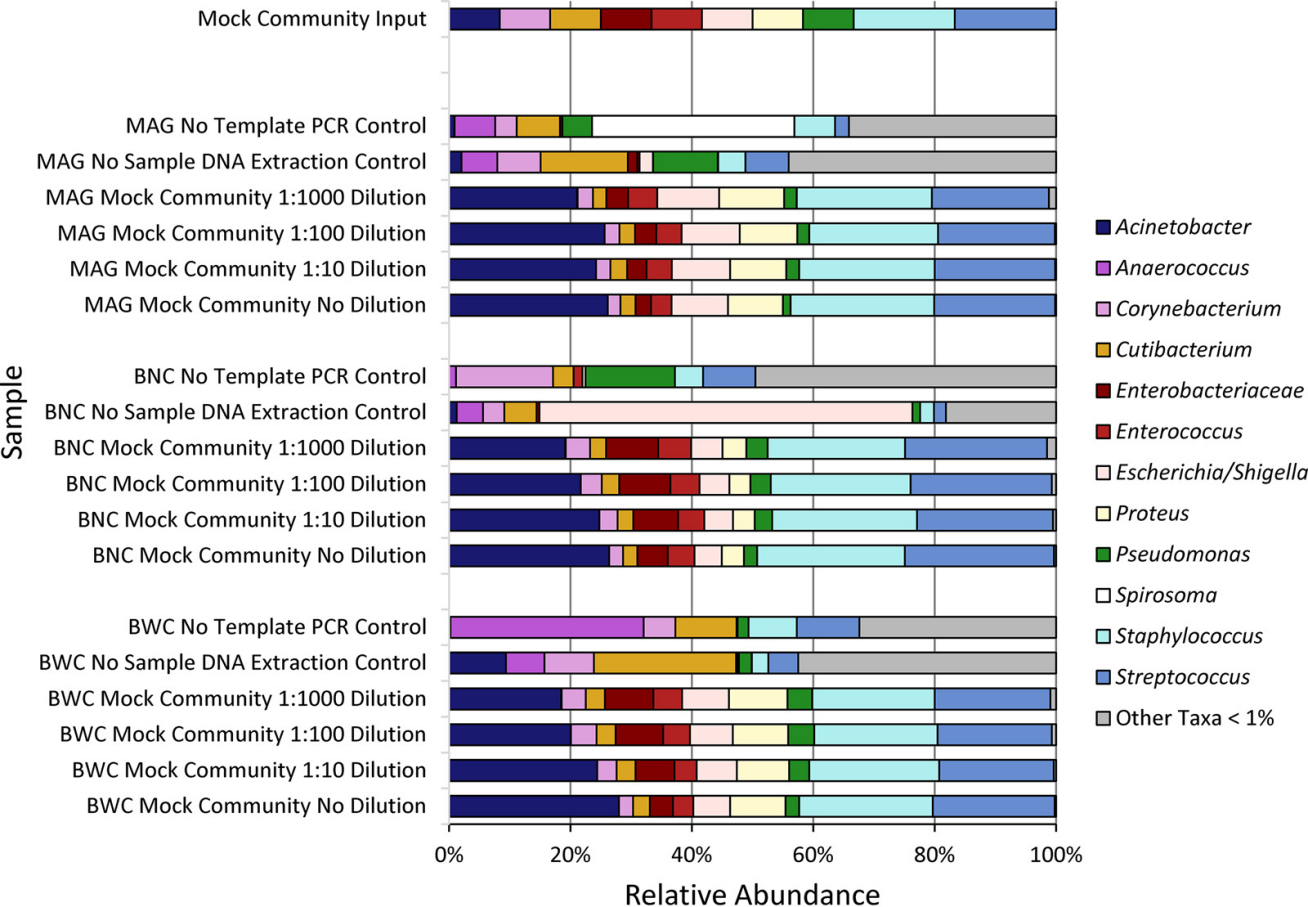
Mock community taxonomic profiles from V4 HTS are overall similar regardless of dilution or DNA extraction method. Shown are bar plots displaying the relative abundances of bacteria identified by sequencing of mock communities according to extraction method and dilution of the input mock community. “Mock community input,” the known composition of the original mock community. MAG, AGOWA Mag Mini DNA isolation kit; BNC, BiOstic bacteremia DNA kit without carrier RNA; BWC, BNC with carrier RNA (which can increase DNA extraction yield). The DNA from mock community cells was extracted using MO BIO's BiOstic Bacteremia DNA kit without carrier RNA (BNC), MO BIO's BiOstic Bacteremia DNA kit with carrier RNA (BWC), and the AGOWA mag Mini DNA isolation kit (MAG). Sequence quality filter and analyses were carried out using the denoising program DADA2. Sequence taxonomy was classified through the SILVA 16S rRNA database.

To characterize the contribution of DNA extraction efficiency and bias, we extracted DNA from this mock community using two different commercially available extraction kits, the AGOWA mag Mini DNA isolation kit (MAG) and BiOstic Bacteremia DNA isolation kit either without (BNC) or with (BWC) carrier RNA, which can increase DNA extraction yield for some kits (10) (Fig. 3). We then compared extraction efficiency and the sequencing-based relative abundance of mock community members among these three processing methods, relative to the known composition of the mock community, within four different dilutions of the mock community, to determine the effects of bacterial load (0- to 1,000-fold), and including controls to identify the bacterial DNA contaminating extraction and PCR reagents.

As shown in Fig. 3, these three methods, followed by 16S HTS, generated overall similar microbiota profiles that reflected the mock community composition to varying degrees, but never perfectly, indicating the bias introduced by sequencing library construction. For example, the relative abundance of Acinetobacter in sequencing results averaged 23%, with an input relative abundance of 8%, while the results for Pseudomonas were 3% and 8%, respectively. Given the overall similarity in results from the three DNA extraction methods (Fig. 3), the bias is likely to be attributable to that known to result from the primers used to amplify a portion of the 16S rRNA gene, V4, which is used commonly for this type of analysis (11). Notably, this primer set and gene variable region are often used for this type of analysis because they are generally capable of genus-level taxonomic identification of bacteria, as was the case here for all mock community members other than Klebsiella (identified at the family level, “Enterobacteriaceae,” and verified by higher-resolution sequencing of this species).

**FIG 4:**
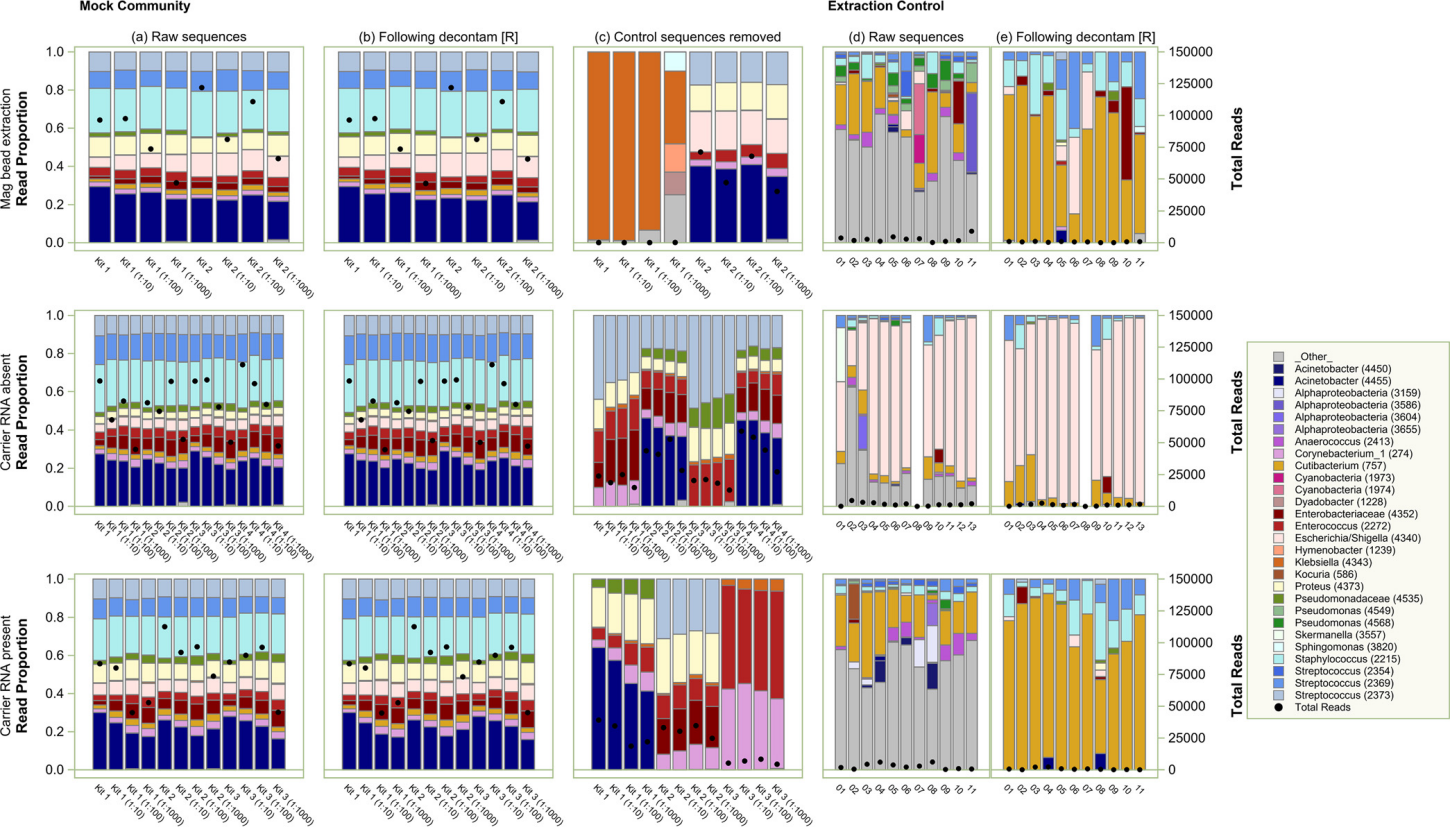
Effect of DNA extraction method and computational decontaminant removal on taxonomic composition of mock community and negative-control samples. Stacked bar-chart results showing relative abundances of taxa after each extraction method and HTS, followed by contaminant sequence removal. Left, profiles shown were generated with (a) unfiltered sequence data; (b) those data after decontam R; (c) after computational removal of all sequences identified in concurrent no-sample extraction controls. Right, profiles from the controls themselves before (d) and after (e) decontam R. We recommend the application of the computational package decontam R to sequences derived from any of the three extraction techniques.

Based on the similarity of the mock community composition before and after processing, we turned our attention to the computational management of contaminant sequences, comparing postprocessing using decontam R analysis (Fig. 4, column b) and computational removal of all sequences identified in concurrent no-sample extraction controls (Fig. 4, column c). We recommend the use of any of the three extraction methods and postprocessing using decontam R to maximize DNA yield and minimize bias (Fig. 4, columns a and b). Full removal led to exclusion of sequences that were known to be in the mock community, and decontam R qualitatively improved concordance with the mock community composition.

### Refined 16S HTS on CSF samples obtained from children diagnosed with bacterial meningitis (objective 2).

To further investigate the utility of computational filtering of contaminant reads, we then applied all three DNA extraction methods and identical postsequence analyses to the CSF samples from children diagnosed with meningitis. We analyzed CSF collected from 22 pediatric meningitis episodes. Among the study population, 59% (*n* = 13) were female, with a median age of 0.6 months (interquartile range [IQR] 0.2 to 0.9) and median gestational age of 39 weeks (IQR 35 to 40).

**FIG 5:**
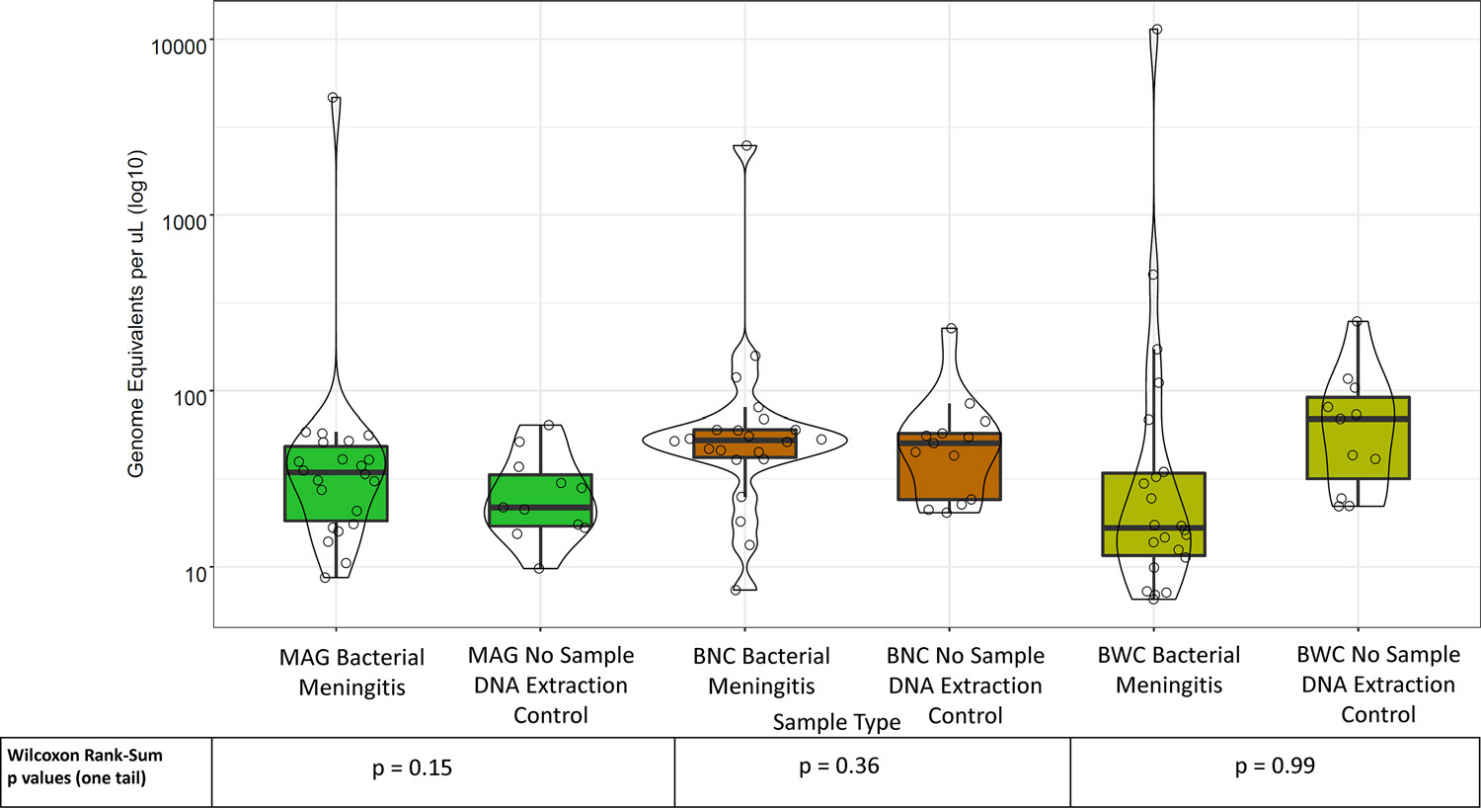
The bacterial DNA concentrations in CSF bacterial meningitis samples are comparable to bacterial DNA concentrations in no-template extraction controls. Box and violin plots of total bacterial abundance in CSF bacterial meningitis samples and their corresponding, concurrent extraction controls by 16S rRNA gene copies measured by qPCR, expressed as genome equivalents per μL (GE/μL).

Before sequencing, we defined the number of bacterial genome equivalents (GEs) recovered from all clinical CSF samples and controls after all three DNA extraction methods, using broad-range 16S rRNA gene quantitative PCR (qPCR, Fig. 5). We found that the amount of bacterial DNA extracted from clinical samples generally did not exceed that from negative controls regardless of processing method. These results suggested that the bacterial load in the CSF samples was relatively low compared with many clinical and environmental samples (5), raising the possibility that our approach (and perhaps any current sequencing-based approach) may not be able to discriminate true pathogens from reagent contaminants.

**FIG 6:**
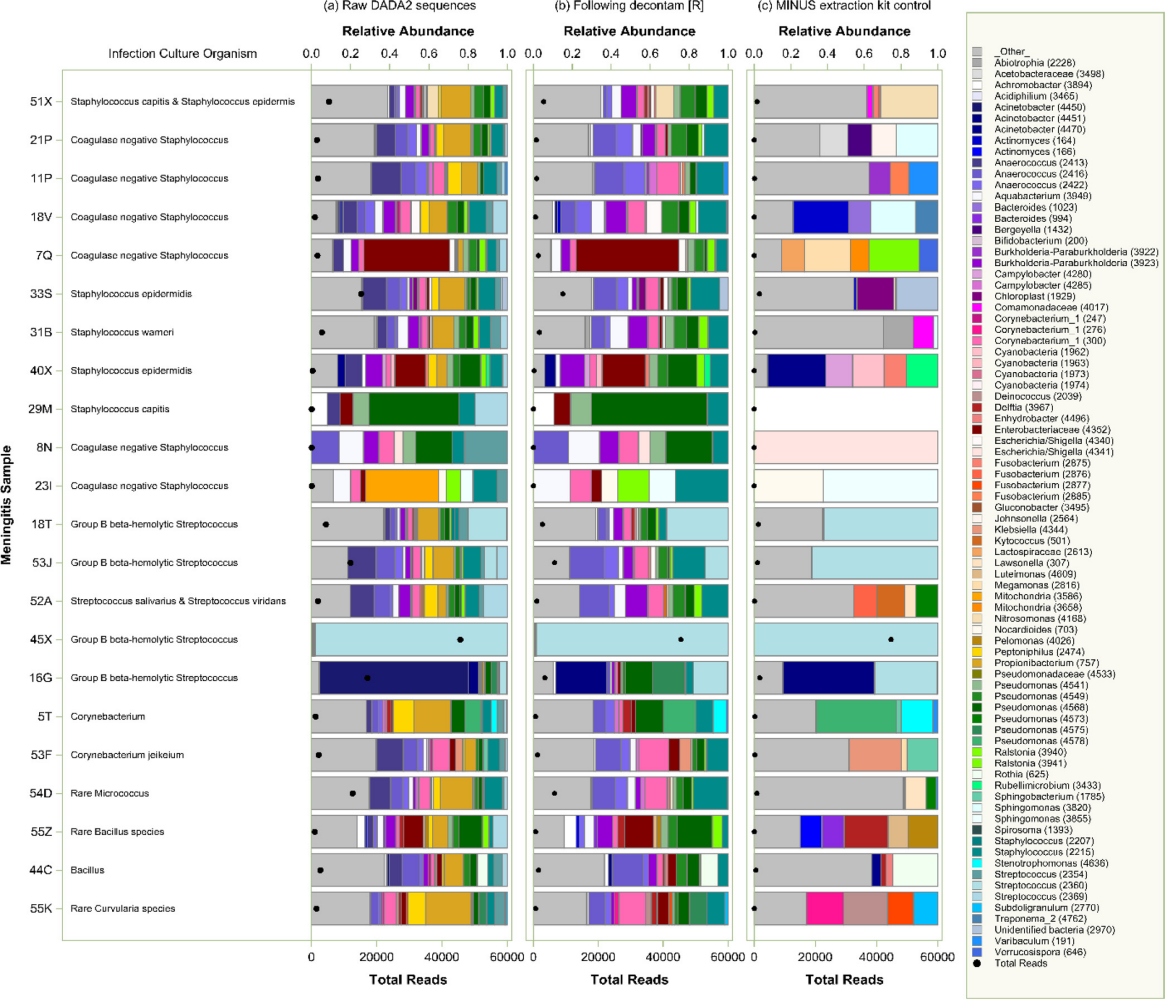
16S HTS of CSF samples from children diagnosed with meningitis, followed by computational filtering of contaminant reads, does not reliably identify culturable bacteria in those samples. CSF samples from children with meningitis are identified by sample ID (left), where the organisms cultured from those samples at diagnosis are indicated. Relative abundances of taxa by genus in raw, unfiltered data using MAG (a) generally changed little with analysis by decontam R (b). While the further complete removal of reads in concurrent no-sample extraction (c) did occasionally enrich for the cultured genus (e.g., 53J), this approach more often did not reliably identify the cultured organism either at all (e.g., 51X, 21P, 11P, 18V, 7Q) or as a dominant constituent (e.g., 16G).

Sequencing resulted in a total of 4,884 unique V4 sequences, of which 249 were identified as likely contaminants and removed. As shown in Fig. 6, while this pipeline did often identify reads of the genera cultured from infection samples at the time of diagnosis, this approach did not reliably indicate the dominance, or even presence, of those cultured organisms. In only 3 out of 22 samples was the cultured genus identified as the dominant organism by 16S HTS (23I, Staphylococcus; 18T and 45X, Streptococcus). In 4 samples, the cultured organism was not identified by 16S HTS at all. Therefore, we concluded that, even with computational filtering of likely or definite contaminant reads, refined 16S HTS was unable to consistently identify infecting pathogens in CSF samples from children with meningitis.

## DISCUSSION

This study sought to optimize the performance of 16S HTS in CSF samples with low bacterial loads by defining and addressing potential sources of error (including the contributions of reagent contamination, variable DNA extraction efficiency and bias, and batch effect). We demonstrated optimal similarity of sequencing-based mock community microbiota profiles and maximization of DNA yield, albeit with some previously observed efficiency bias, with all three extraction techniques and postprocessing using decontam R at the low bacterial loads observed in CSF samples. We then applied these methods to CSF samples from children with bacterial meningitis. We demonstrated that, even with a rigorously controlled sample processing and 16S HTS sequencing approach, augmented by computational removal of contaminant sequencing signal, we were unable to reliably identify infecting organisms identified by culture in CSF samples with low bacterial loads from children with clinically diagnosed meningitis. Due to the low abundance of organisms in these samples relative to that of DNA in most commercially available processing reagents, the “signal” from infecting bacteria could not be distinguished from the “background noise” of contaminants. Therefore, without major methodologic advances, culture currently remains the best approach to identifying pathogens infecting in this clinical context. Our hypothesis that, unlike CSF shunt infection, there would be high levels of bacteria in bacterial meningitis samples was disproven.

16S HTS and other molecular microbiological techniques have proven to both complement and, in some cases, show promise for replacing culture in other clinical scenarios, usually where bacterial abundances are much higher than that of contaminants. For example, sequencing-based microbiome methods are in common use for studying the microbiota of the gastrointestinal tract, vagina, mouth, and other exposed mucosal surfaces. In addition, culture is frequently unable to identify pathogenic organisms in patients with meningitis, despite the clear purulence of the resulting samples (12). Therefore, there is great interest in using culture-independent, DNA-based methods for pathogen identification in CSF samples (12, 13). In this study, we found that the nearly consistent low bacterial abundances relative to the contamination levels of reagents greatly diminish the utility of molecular approaches, even with the inclusion of rigorous controls and computational removal of contaminant sequencing reads, regardless of how the samples are processed or extracted.

A panel of experts previously performed a narrative review to describe the desirable characteristics for a molecular diagnostic assay for bacterial meningitis (14). The authors concluded that such an assay would detect multiple pathogens in a cost-effective, easy-to-use system that provides rapid and robust results. Unfortunately, our analysis indicates that, absent major methodologic advances, such an 16S HTS assay does not currently exist. However, current molecular methods are already in common use for characterizing higher-abundance samples, such as purulent respiratory infections (15), gastrointestinal infections (16), and even normally sterile fluids such as blood (17). In contrast, infections with relatively low abundances of microbes in the resulting samples pose considerable challenges to these methods. As numerous authors have reviewed (4, 6, 18 to 24), the uncertainty and error introduced by reagents, human error, and methodologic bias renders current methodology concerningly inaccurate, in some cases leading to controversial or variable study results. Indeed, investigators have reached conclusions similar to those here for studying nasopharyngeal samples and induced sputum, which can also exhibit low bacterial loads (25).

Our results contrast with prior studies by others investigating the utility of 16S HTS for diagnosing or identifying pathogens in children and adults with meningitis (20 to 24, 26, 27). While comprehensively contrasting our approach with these others is beyond the scope of the manuscript, two of these studies defined detection of specific pathogens as exceeding a limit of 4 reads in the case of Streptococcus pneumoniae (22) and 19 reads for any pathogen (23), respectively, raising questions regarding whether these limits exceeded the background signal from reagents. In general, these studies did not quantify the effects of sample extraction or reagent contaminants, precluding direct comparison with our approaches here.

We previously used 16S HTS to identify bacterial DNA reads in CSF from children with shunt infections (28), where the bacterial loads in infection samples often exceeded 10^5^ CFU/mL. While we have refined the technique of 16S HTS for use in CSF and demonstrated its limited utility in bacterial meningitis, we believe this approach may be more useful for CSF shunt infection, where persistence of bacteria on hardware may increase sensitivity of molecular diagnostics relative to that for meningitis. Together with the results of the current study, these findings highlight the possible role of restricting molecular microbiological analysis to cases where loads exceed the limits of detection, which must be defined precisely for the specific analytical pipeline being used. We also propose that a screening qPCR for total bacterial load, performed concurrently with culture, could be a useful step prior to considering the utility of HTS for clinical diagnosis.

Our study had a number of limitations. For example, our CSF sample set originated from a single geographic area and reflected a limited sample size (*n* = 22), restricting generalizability. In addition, the volume of CSF available for our analyses was relatively low, 100 μL, which could conceivably have limited our sensitivity. Other DNA extraction methods could have improved sensitivity, an issue we attempted to address by comparing 3 different such methods. Similarly, work with fecal samples indicates that qPCR has limitations in quantifying total bacterial load in some contexts, and this issue may have impacted our results (29, 30). While we constructed a mock community to optimize conditions, with hindsight it should have been diluted further to approach the low bacterial load of CSF. It is also likely that variation in 16S rRNA gene copy number between bacterial species impacted our findings, an issue for which a consistently useful approach has not yet been found. Despite these limitations, we believe we have comprehensively tested conditions for 16S HTS testing of low bacterial load clinical samples such as CSF.

In conclusion, we demonstrated optimal similarity in microbiota profiles yielded by sequencing of a mock community and maximization of DNA yield, albeit with some previously observed efficiency bias, after three extraction techniques and postprocessing using decontam R under the low bacterial load conditions seen in CSF samples. We found that the limits of detection imposed by reagents and methodologic bias precluded the accurate detection of infecting bacteria in children with culture-confirmed meningitis using 16S HTS despite rigorous controls and sophisticated computational approaches. Although we did not find current DNA-based diagnostics to be useful for meningitis, the utility of these methods to detect CSF shunt infection remains undefined. Future advances in sample processing methods to minimize or eliminate contamination will be required to improve the sensitivity and specificity of these methods for pediatric meningitis.

## MATERIALS AND METHODS

### Objective 1 (optimize the performance of 16S HTS in CSF samples with low bacterial loads).

We used laboratory techniques to control systematic error associated with reagent contamination, DNA extraction efficiency and bias, and batch effects, comparing three such bench pipelines when extracting an experimentally constructed suspension of bacteria commonly infecting CSF (a “mock community”). Two computational methods were then used to remove potential contaminant sequences from the resulting 16S HTS results. The refined approach was then applied to bacterial meningitis samples (Fig. 1). More complete details of each of these approaches are provided as follows.

To minimize the likelihood of cross-contamination between mock community samples, controls, and CSF study samples, DNA extraction and sequencing library construction were performed physically and temporally separate for each of these three sample types for all experiments.

### Mock community.

The mock community was created by mixing bacterial DNA extracted from equivalent cell numbers of strains of bacteria known to be frequently isolated from CSF shunt infections (31) (Table 1), yielding a total final concentration of 6.00 × 10^7^ CFU equivalents/μL. Serial 1:10 dilutions were made of this mock community, ranging from 6.00 × 10^7^ CFU equivalents/μL to 6.00 ×10^4^ CFU equivalents/μL. All bacteria used for mock communities were cultured at 37°C on LB agar plates, except for Staphylococcus aureus, which was cultured on blood agar, and Cutibacterium acnes, cultured anaerobically on blood agar. DNA from each culture was extracted using the CTAB DNA extraction method as described in “Bacterial genomic DNA isolation using CTAB” (https://jgi.doe.gov/wp-content/uploads/2014/02/JGI-Bacterial-DNA-isolation-CTAB-Protocol-2012.pdf). Bacteria were harvested from the solid agar media and resuspended in 3 mL of Tris-EDTA (TE) buffer and adjusted to OD_600_ 1.0. From this suspension, 740 μL was transferred to a clean centrifuge tube, and DNA concentration for each was measured using the Qubit dsDNA broad-range (BR) assay. CFU equivalents/μL were calculated for each using the following calculation: CFU equivalents/μL = (DNA concentration [ng/μL] × [6.022E + 23])/(length of template [bp] × [1.00E + 09] × 650).

DNA concentration was adjusted for each individual member of the mock community to the equivalent of 1.00E + 07 CFU equivalents/μL in a total volume of 50 μL. These were then combined to a total volume of 550 μL with an estimated total bacterial load of 1.10E + 08 CFU equivalents/μL. Aliquots of this mock community were stored at −80°C until required, when stocks were thawed on ice and serially diluted to the desired total bacteria loads in a total of 100 μL and processed through the indicated DNA extraction protocols using the same steps as with the CSF samples and controls, with the exception of bead-beating.

We used this mock community to optimize and compare our sample processing and sequencing procedures prior to analyzing patient CSF samples and to identify and address biases introduced during DNA extraction, library construction, and taxonomic assignment. A total of 36 mock community samples (including dilutions) were included in our experiments comparing results of three different DNA extraction protocols (Table 2).

**TABLE 2:** Number of control samples used for each DNA extraction type and for library PCR

Controls		DNA extraction method			Total
	Purpose	Mag mini kit (MAG) (LGC, Biosearch Technologies, Hoddesdon, UK)	BiOstic bacteremia DNA isolation kit (BNC) (Qiagen Maryland United States)	BiOstic bacteremia DNA isolation kit with carrier RNA (BWC) (Qiagen, Maryland, USA)	
Mock community	Identification of possible biases introduced during DNA extraction, library construction, and taxonomic assignment	8^*a*^	16^*a*^	12^*a*^	36
No-sample DNA extraction controls	Identification of potential contaminants from kit components	11^*b*^	13^*b*^	11^*b*^	35
The sample no-template library PCR controls	Performed in parallel with the CSF sample library PCRs Identification of potential contaminants during generation of sample 16S rRNA libraries	3	3	3	9^*c*^
The mock community no-template library PCR controls	No-template library PCR controls performed in parallel with the Mock Community library PCRs Identification of potential contaminants during generation of 16S rRNA Mock community libraries				4^*c*^
The no-sample DNA extraction controls No-template library PCR controls	No-template library PCR controls performed in parallel with the No-sample DNA extraction controls library PCRs Identification of potential contaminants during generation of 16S rRNA libraries				5^*c*^
Sequencing negative control	An unused index ID included in the sequence data sheet. Identification of mis-assigned reads during sequencing analysis				1
Total					90

a Number of mock community controls per method varied depending on number of kits used to extract the sample set, for required technical replicates, and to address day-to-day variability.

b Number of no-sample DNA extraction controls per method depended on number of kits used to extract the sample set and required technical replicates, which differed between methods.

c Total number of no-template library PCR control includes three such controls performed for each DNA extraction method (nine in total), four for the mock community analysis, and five for the no-sample DNA extraction controls.

### Control samples.

In addition to mock communities, each mock community analysis included several control samples to identify and quantify bacterial DNA contamination in DNA extraction kits and PCR reagents (reagent contamination). These controls included (i) samples in which the processing reagents were included, with no purposely input (“template”) DNA, from DNA extraction and before the PCR step required to construct sequencing libraries (“no-sample DNA extraction control”), and (ii) other samples with only the reagents used during and after PCR and library construction (“no-template library PCR control”). In both types of controls, water was used to make up for the sample volumes of CSF and mock community extraction experiments. A total of 35 no-template extraction controls were included in DNA extraction experiments, and 18 no-template controls were included in library experiments. (Table 2). The PCR for a group was repeated if the no-template control yielded a visible EtBr-stained band on a 1% agarose gel. Otherwise, CSF sample amplicons and no-template controls were analyzed using identical protocols.

### DNA extraction.

To address methodologic variation in DNA extraction efficiency and bias, DNA was extracted and purified from all samples using three extraction approaches: the AGOWA Mag Mini DNA isolation kit (LGC Genomics, Berlin, Germany), here MAG; the BiOstic Bacteremia DNA isolation kit (Qiagen), here BNC; and BNC with the inclusion of carrier RNA (Qiagen) to increase DNA yield by preventing binding to plastic in the kits (10), here BWC. Components of each kit were aliquoted before extraction in an AirClean Systems PCR Workstation (USA Scientific) decontaminated with LookOut DNA Erase (Sigma-Aldrich), according to the manufacturer’s instructions, followed by 15 min of UV-ray exposure to minimize environmental contamination. The molecular-grade water used in all analyses originated from the same 500-mL bottle that had been previously aliquoted into 15 mL lots, and was frozen at −20°C and thawed at room temperature for use as required, with each step performed in the PCR workstation described above to minimize contamination.

MAG extractions were performed as follows without carrier RNA due to manufacturer concerns that this RNA could displace sample DNA during extraction (Heiko Hauser, Head of R&D for LGC Group, personal communication). A 100-μL volume of each sample was aliquoted into a sterile low binding microfuge tube (Axygen, catalogue number [CN] 31104081), to which 20 μL of 20 mg/mL Proteinase K (Invitrogen, CN 25530-049) was added. The mixture was vortexed for 20 s and incubated at 55°C for 10 min. After incubation, 250 μL of lysis buffer was added to the tube and vortexed gently for 15 s. The mixture was transferred to a clean 2-mL tube (Sarstedt, CN 72.693.005) containing 0.3 g of 0.1 mm zirconia/silica beads (Biospec Products, Bartlesville, OK, USA [Biospec], CN 11079101z). Using a Mini-Beadbeater-16 (Biospec, CN 607), the sample was mechanically disrupted by bead-beating for 3 min, followed by a 1-min incubation on ice and a further 3 min of bead-beating. The sample was centrifuged at 4,000 rpm for 10 min. The resulting supernatant was transferred to a new low binding microfuge tube. To this, 325 μL of binding buffer and 10 μL of mag particle suspension (mag-particles) were added, vortexed for 15 s, and incubated at room temperature for 30 min with continuous mixing on a VWR Tube Rotator (VWR, CN 10136-084). After the incubation step, DNA extraction proceeded according to the manufacturer’s instructions as described (28).

In both BIOstic extraction methods, 100 μL of sample was mixed by gentle vortexing either with or without (depending on the method) 1 μL of added carrier RNA at a stock concentration of 1 μg/μL of RNA. DNA was then extracted from each sample according to the manufacturer’s instructions and as described (28).

### Batch effects.

To control for batch effects, all CSF samples were randomized using a random number generator program (32). Extractions were performed by research staff blinded to the sample identification key.

### Bacterial quantification.

A quantitative PCR (qPCR) assay targeting the 16S rRNA genes was used to measure the total bacterial load in each CSF sample as described earlier (28). The number of genome equivalents (where one GE = 1 bacterial genome) of bacteria per mL (GE/mL) of CSF was calculated using values estimated from the 16s rRNA qPCR assay and from sample volumes from DNA extraction using the following formula:

$$\text{GE}/\text{mL }=\;\left(\left(\left({\text{GE}}_{\text{well}}/{\text{TMP}}_{\text{vol}}\right){\text{ × EL}}_{\text{vol}}\right)/{\text{EX}}_{\text{vol}}\right)$$

where:

GE_well_ = genome equivalents per reaction well (estimated from the standard curve)

TMP_vol._ = volume of extracted DNA template added to the reaction

EL_vol_ = original DNA elution volume from extraction

EX_vol_ = volume CSF sample used in DNA extraction

### Bacterial 16S rRNA gene amplification, sequencing, and analyses (16S HTS).

Amplicon library construction was carried out using a one-step PCR amplification targeting the 16S rRNA gene V4 region (33). The amplicon library primers (34) each contained the specific Illumina adapters, an 8-bp index sequence to allow for multiplex sequencing of the samples, and the 16S rRNA gene-specific primer (33). A one-step approach that combines both 16S rRNA gene amplification and the addition of adapter and index sequences was used to minimize risks of both cross-contamination and formation of chimeric amplicons. Library construction, pooling, and sequencing proceeded as described for 600 cycles on an Illumina MiSeq desktop sequencer using the Miseq reagent kit v. 3 (28). Index hopping was minimized by unique dual indexing, a strategy shown to minimize sample index swaps (35), as well as use of the non-patterned MiSeq rather than patterned Illumina flowcells (36).

### 16S HTS analysis.

Sequencing data were processed using the denoising program DADA2 (37) (v. 1.6.0) as described (38), and aligned to the SILVA 16S reference database (v. 132) (39) to produce a 16S-amplicon taxa table for downstream computational analysis. All samples, regardless of sample type or extraction protocol, were run on the same Illumina flowcell to reduce interrun variation.

### Postsequencing contaminant removal.

Two analytic strategies were used to identify and remove contaminant sequences. Using the decontam R package installed from GitHub (https://github.com/benjjneb/decontam) (40), contaminant sequences were identified as those *either* with decreasing abundance at higher concentrations (threshold *P* < 0.10) *or* that were more prevalent in kit control samples than in CSF samples (threshold *P* < 0.50). Additionally, all sequences detected in extraction kit controls were considered contaminants and were removed from the CSF samples. Results from each computational approach were compared with the known mock community composition for objective 1 and microbiological culture results for objective 2.

### Objective 2 (perform refined 16S HTS on CSF samples from bacterial meningitis).

**(i) Study subjects.** Children ≤18 years old undergoing treatment for conventional culture-confirmed meningitis at St. Louis Children’s Hospital in St. Louis, Missouri were eligible for enrollment in this study. Enrollment occurred from 2009 to 2014. Meningitis was defined as identification of organisms on microbiological culture of CSF fluid obtained from a lumbar puncture in a child without an existing neurosurgical device. All study subjects’ CSF underwent routine microbiological processing and testing in the St. Louis Children’s Hospital Microbiology Laboratory. For this study, we examined CSF obtained from the subset of 40 children whose conventional microbiological cultures recovered bacterial organisms and for whom there was 300 μL of banked CSF available. The study received Institutional Review Board approval from the Seattle Children’s Research Institute, Washington University at St. Louis, and Children’s Hospital Los Angeles.

**(ii) Clinical data.** For each child, we collected culture information, specifically organism recovered in CSF and blood if applicable.

**(iii) CSF specimen collection.** Sterile conditions were standard practice throughout recovery and storage of CSF. CSF samples were acquired via lumbar puncture under sterile conditions for clinical purposes and transferred to the St. Louis Children’s Hospital clinical laboratory. The clinical laboratory performed microbiological cultures, and samples were frozen at −20°C for 3 months. They were then transported on ice to the Washington University Neonatal CSF Repository, where they were stored at −80°C prior to being shipped overnight on dry ice for analysis.

**(iv) Conventional microbiological culture identification of bacteria.** All CSF samples were tested using routine aerobic culture techniques in hospital-certified laboratories at St. Louis Children’s Hospital. Conventional cultures are the traditional diagnostic approach used to detect typical pathogens in infectious diseases and were performed in a clinical microbiology laboratory following Clinical and Laboratory Standards Institute guidelines; however, conventional culture approaches do not detect all bacteria present in human disease (41, 42). During analysis, the laboratory team remained blinded to the samples’ culture results.

**(v) Statistical analyses.** Samples were analyzed as described above for objective 1 mock samples. Sequence prevalence was calculated for each sample as the ratio of sequence reads divided by total reads. While sequences have been linked to genera to aid in clinical interpretation, multiple sequences may be associated with the same organism or with unique variants; therefore, the sum of sequence reads associated with a unique genus does not necessarily equal the frequency of that genus in the sample prevalence. For visual clarity, sequences representing less than 10% of reads across all samples, extraction methods, and postprocessing steps were aggregated to a single “other” category in figures. A principal coordinates analysis (PCoA) based on Bray–Curtis distances was performed at the genus level to assess species composition dissimilarities between no-sample DNA extraction controls, no-template PCR controls, and the mock communities. Permutational multivariate analysis of variance (PERMANOVA) (“Adonis” function, vegan package, R; 1,000 permutations) was used to assess the influence of sample type on the microbiota populations.

### Data availability.

The data sets generated and/or analyzed during the current study are available in the NCBI Sequence Read Archive repository, https://www.ncbi.nlm.nih.gov/sra as PRJNA768849: “A refined, controlled 16S rRNA gene sequencing approach reveals limited detection of cerebrospinal fluid microbiota in children with bacterial meningitis.”
